# Methyl (*E*)-2-({2-[(*E*)-(hy­droxy­imino)­meth­yl]phen­oxy}meth­yl)-3-phenyl­acrylate

**DOI:** 10.1107/S1600536812002711

**Published:** 2012-02-04

**Authors:** G. Suresh, V. Sabari, J. Srinivasan, Bakthadoss Mannickam, S. Aravindhan

**Affiliations:** a Department of Physics, Presidency College, Chennai 600 005, India; b Department of Organic Chemistry, University of Madras, Chennai 600 025, India

## Abstract

In the title compound, C_18_H_17_NO_4_, the hy­droxy­ethanimine group is essentially coplanar with the ring to which it is attached [C—C—N—O torsion angle = 179.94 (14)°]. The mol­ecules are linked into cyclic centrosymmetric *R*_2_^2^(6) dimers *via* O—H⋯N hydrogen bonds and the crystal packing is further stabilized by C—H⋯O inter­actions.

## Related literature

For structures of other acrylate derivatives, see: Zhang *et al.* (2009 ▶); Wang *et al.* (2011 ▶); SakthiMurugesan *et al.* (2011 ▶); Govindan *et al.* (2011 ▶). For the use of oxime ligands in coordination chemistry, see: Chaudhuri (2003 ▶). For the biological activity of caffeic acids, see: Hwang *et al.* (2001 ▶); Altug *et al.* (2008 ▶); Ates *et al.* (2006 ▶); Atik *et al.* (2006 ▶); Padinchare *et al.* (2001 ▶).
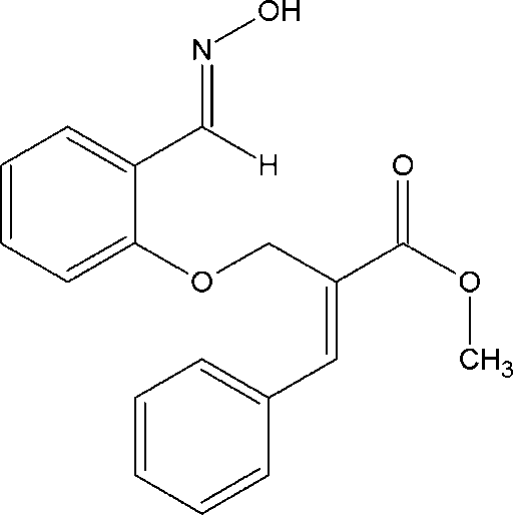


## Experimental

### 

#### Crystal data

C_18_H_17_NO_4_
*M* *_r_* = 311.33
Monoclinic, 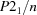

*a* = 9.6463 (4) Å
*b* = 7.7062 (3) Å
*c* = 22.4675 (9) Å
β = 100.337 (2)°
*V* = 1643.04 (11) Å^3^
*Z* = 4
Mo *K*α radiation
μ = 0.09 mm^−1^
*T* = 293 K
0.2 × 0.2 × 0.2 mm

#### Data collection

Oxford Diffraction Xcalibur-S diffractometer
Absorption correction: multi-scan (*CrysAlis PRO*; Oxford Diffraction, 2009 ▶) *T*_min_ = 0.980, *T*_max_ = 0.990
17182 measured reflections
3692 independent reflections
2537 reflections with *I* > 2σ(*I*)
*R*_int_ = 0.029

#### Refinement

*R*[*F* ^2^ > 2σ(*F* ^2^)] = 0.046
*wR*(*F* ^2^) = 0.127
*S* = 1.02
3692 reflections
211 parameters
H-atom parameters constrained
Δρ_max_ = 0.21 e Å^−3^
Δρ_min_ = −0.18 e Å^−3^

### 

Data collection: *CrysAlis PRO* (Oxford Diffraction, 2009 ▶); cell refinement: *CrysAlis PRO*; data reduction: *CrysAlis PRO*; program(s) used to solve structure: *SHELXS97* (Sheldrick, 2008 ▶); program(s) used to refine structure: *SHELXL97* (Sheldrick, 2008 ▶); molecular graphics: *ORTEP-3 for Windows* (Farrugia, 1997 ▶); software used to prepare material for publication: *PLATON* (Spek, 2009 ▶).

## Supplementary Material

Crystal structure: contains datablock(s) I, global. DOI: 10.1107/S1600536812002711/bt5745sup1.cif

Structure factors: contains datablock(s) I. DOI: 10.1107/S1600536812002711/bt5745Isup2.hkl

Supplementary material file. DOI: 10.1107/S1600536812002711/bt5745Isup3.cml

Additional supplementary materials:  crystallographic information; 3D view; checkCIF report

## Figures and Tables

**Table 1:** Hydrogen-bond geometry (Å, °)

*D*—H⋯*A*	*D*—H	H⋯*A*	*D*⋯*A*	*D*—H⋯*A*
O1—H1*A*⋯N1^i^	0.82	2.06	2.7836 (19)	146
C15—H15⋯O3^ii^	0.93	2.53	3.300 (2)	140

Symmetry codes: (i) 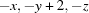; (ii) 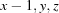.

**Fig. 1.**
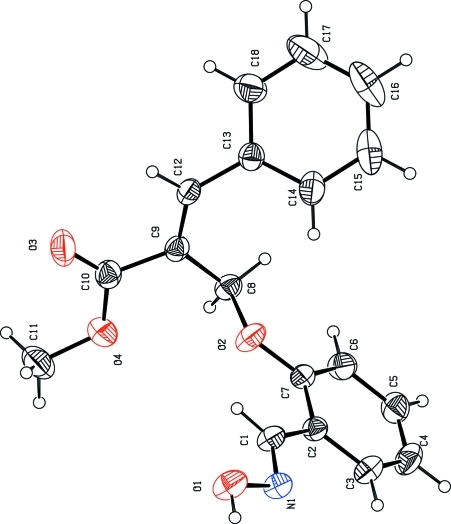
The molecular structure of the title compound, showing 30% probability displacement ellipsoids for H atoms. H atoms have been omitted for clarity.

**Fig. 2.**
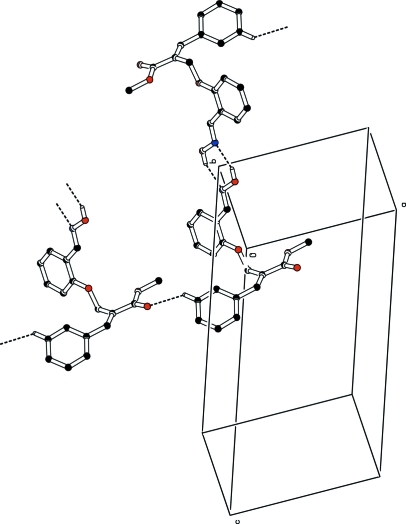
A view of the crystal packing. H atoms not involved in hydrogen bonding (dashed lines) have been omitted for clarity.

## supplementary crystallographic information

### Comment

Recently, 2-cyanoacrylates have been extensively used as agrochemicals because of their unique mechanism of action and good environmental profiles (Zhang *et al.*, 2009). Oximes are a classical type of chelating ligands which are widely used in coordination and analytical chemistry (Chaudhuri, 2003). Some naturally occurring caffeic acids and their esters attract much attention in biology and medicine (Hwang *et al.*, 2001; Altug *et al.*, 2008).These compounds show antiviral, antibacterial, vasoactive, antiatherogenic, antiproliferative, antioxidant and antiinflammatory properties (Atik *et al.*,2006; Padinchare *et al.*, 2001; Ates *et al.*, 2006). Against this background,and in order to obtain detailed information on molecular conformations in the solid state, an X-ray study of the title compound was carried out and the results are presented here. X-Ray analysis confirms the molecular structure and atom connectivity as illustrated in Fig. 1. The oxime group having the C=N forming an E configuration. The hydroxy ethanimine group is essentially coplanar with the ring to which it is attached [C2—C1—N1—O1 torsion angle = 179.9°]

The enoate group assumes an extended conformation as can be seen from torsion angles C12—C9—C10—O4 [-178.7°] and C9—C10—O4—C11 [178.4°]. The hydroxy ethanimine group in the molecules are linked into cyclic centrosymmetric dimers *via* O—H···N hydrogen bonds with the motif *R*_2_^2^(6)(Wang *et al.* (2011), Govindan *et al.* (2011), SakthiMurugesan *et al.* (2011)). Crystal packing is stabilized by C15—H15···O3 and O1—H1A···N1 type intermolecular hydrogen bonds and values are tabulated. The crystal packing (Fig.2) shows the presence of inter-molecular hydrogen bonding.

### Experimental

To a stirred solution of (*E*)-methyl 2-((2-formylphenoxy)methyl)-3-phenylacrylate (4 mmol) in 10 ml of EtOH/H_2_O mixture (1:1) was added NH_2_OH.HCl (6 mmol) in the presence of 50% NaOH at room temperature. Then the reaction mixture was allowed to stir at room temperature for 1.5 h. After completion of the reaction, solvent was removed and crude mass was diluted with water (15 ml) and extracted with ethyl acetate (3 × 15 ml). The combined organic layer was washed with brine (2 × 10 ml) and dried over anhydrous Na_2_SO_4_ and then evaporated under reduced pressure to obtain (*E*)-Methyl 2-((2-((*E*)-(hydroxyimino)methyl)phenoxy)methyl)-3-phenylacrylate as a colourless solid.

### Refinement

Hydrogen atoms were set to calculated positions and refined as riding on their parent atoms with O-H = 0.82Å and C-H ranging from 0.93Å to 0.97Å and U(H) set to 1.2 U_eq_(C) or 1.5 U_eq_(C_methyl_, O).

### Crystal data

**Table:**

C_18_H_17_NO_4_	*F*(000) = 656
*M**_r_* = 311.33	*D*_x_ = 1.259 Mg m^−^^3^
Monoclinic, *P*2_1_/*n*	Mo *K*α radiation, λ = 0.71073 Å
*a* = 9.6463 (4) Å	Cell parameters from 8725 reflections
*b* = 7.7062 (3) Å	θ = 2.8–29.1°
*c* = 22.4675 (9) Å	µ = 0.09 mm^−^^1^
β = 100.337 (2)°	*T* = 293 K
*V* = 1643.04 (11) Å^3^	Monoclinic, colourless
*Z* = 4	0.2 × 0.2 × 0.2 mm

### Data collection

**Table:**

Oxford Diffraction Xcalibur-S diffractometer	3692 independent reflections
Radiation source: fine-focus sealed tube	2537 reflections with *I* > 2σ(*I*)
Graphite monochromator	*R*_int_ = 0.029
Detector resolution: 15.9948 pixels mm^-1^	θ_max_ = 27.3°, θ_min_ = 2.2°
ω scans	*h* = −12→12
Absorption correction: multi-scan (*CrysAlis PRO*; Oxford Diffraction, 2009)	*k* = −9→9
*T*_min_ = 0.980, *T*_max_ = 0.990	*l* = −18→29
17182 measured reflections	

### Refinement

**Table:**

Refinement on *F*^2^	Secondary atom site location: difference Fourier map
Least-squares matrix: full	Hydrogen site location: inferred from neighbouring sites
*R*[*F*^2^ > 2σ(*F*^2^)] = 0.046	H-atom parameters constrained
*wR*(*F*^2^) = 0.127	*w* = 1/[σ^2^(*F*_o_^2^) + (0.0557*P*)^2^ + 0.3308*P*] where *P* = (*F*_o_^2^ + 2*F*_c_^2^)/3
*S* = 1.02	(Δ/σ)_max_ < 0.001
3692 reflections	Δρ_max_ = 0.21 e Å^−^^3^
211 parameters	Δρ_min_ = −0.18 e Å^−^^3^
0 restraints	Extinction correction: *SHELXL97* (Sheldrick, 2008), Fc^*^=kFc[1+0.001xFc^2^λ^3^/sin(2θ)]^-1/4^
Primary atom site location: structure-invariant direct methods	Extinction coefficient: 0.0173 (18)

### Special details

**Table:**

Geometry. All e.s.d.'s (except the e.s.d. in the dihedral angle between two l.s. planes) are estimated using the full covariance matrix. The cell e.s.d.'s are taken into account individually in the estimation of e.s.d.'s in distances, angles and torsion angles; correlations between e.s.d.'s in cell parameters are only used when they are defined by crystal symmetry. An approximate (isotropic) treatment of cell e.s.d.'s is used for estimating e.s.d.'s involving l.s. planes.
Refinement. Refinement of *F*^2^ against ALL reflections. The weighted *R*-factor *wR* and goodness of fit *S* are based on *F*^2^, conventional *R*-factors *R* are based on *F*, with *F* set to zero for negative *F*^2^. The threshold expression of *F*^2^ > σ(*F*^2^) is used only for calculating *R*-factors(gt) *etc*. and is not relevant to the choice of reflections for refinement. *R*-factors based on *F*^2^ are statistically about twice as large as those based on *F*, and *R*- factors based on ALL data will be even larger.

### Fractional atomic coordinates and isotropic or equivalent isotropic displacement parameters (Å^2^)

**Table:**

	*x*	*y*	*z*	*U*_iso_*/*U*_eq_	
C1	0.00948 (17)	0.7108 (2)	0.07286 (7)	0.0513 (4)	
H1	0.0818	0.7020	0.1062	0.062*	
C2	−0.09006 (15)	0.56814 (19)	0.05966 (6)	0.0444 (3)	
C3	−0.20363 (17)	0.5708 (2)	0.01196 (7)	0.0583 (4)	
H3	−0.2181	0.6677	−0.0130	0.070*	
C4	−0.29464 (18)	0.4335 (3)	0.00100 (8)	0.0667 (5)	
H4	−0.3711	0.4384	−0.0307	0.080*	
C5	−0.27291 (18)	0.2889 (3)	0.03682 (8)	0.0626 (5)	
H5	−0.3344	0.1953	0.0290	0.075*	
C6	−0.16062 (18)	0.2804 (2)	0.08442 (8)	0.0566 (4)	
H6	−0.1459	0.1815	0.1084	0.068*	
C7	−0.07031 (15)	0.4206 (2)	0.09599 (6)	0.0449 (4)	
C8	0.08136 (16)	0.2771 (2)	0.17851 (7)	0.0489 (4)	
H8A	0.1178	0.1890	0.1546	0.059*	
H8B	0.0014	0.2294	0.1937	0.059*	
C9	0.19303 (15)	0.33459 (19)	0.22971 (7)	0.0447 (4)	
C10	0.34042 (17)	0.3505 (2)	0.21965 (8)	0.0532 (4)	
C11	0.4897 (2)	0.3269 (3)	0.14769 (11)	0.0870 (7)	
H11A	0.5201	0.4455	0.1522	0.130*	
H11B	0.4865	0.2909	0.1066	0.130*	
H11C	0.5546	0.2546	0.1741	0.130*	
C12	0.16887 (15)	0.37404 (19)	0.28457 (7)	0.0460 (4)	
H12	0.2478	0.4040	0.3129	0.055*	
C13	0.03513 (16)	0.3767 (2)	0.30627 (7)	0.0503 (4)	
C14	−0.09178 (17)	0.4264 (3)	0.27074 (9)	0.0672 (5)	
H14	−0.0935	0.4638	0.2313	0.081*	
C15	−0.2148 (2)	0.4205 (3)	0.29358 (13)	0.0915 (8)	
H15	−0.2995	0.4531	0.2695	0.110*	
C16	−0.2125 (3)	0.3667 (3)	0.35192 (16)	0.1059 (9)	
H16	−0.2963	0.3582	0.3668	0.127*	
C17	−0.0873 (3)	0.3256 (3)	0.38844 (14)	0.1111 (9)	
H17	−0.0856	0.2931	0.4284	0.133*	
C18	0.0351 (2)	0.3325 (3)	0.36579 (10)	0.0784 (6)	
H18	0.1201	0.3070	0.3910	0.094*	
N1	0.00127 (14)	0.84521 (17)	0.04087 (6)	0.0510 (3)	
O1	0.10817 (14)	0.96479 (16)	0.06224 (6)	0.0694 (4)	
H1A	0.1018	1.0483	0.0393	0.104*	
O2	0.04067 (12)	0.42920 (14)	0.14275 (5)	0.0603 (3)	
O3	0.43880 (12)	0.3942 (2)	0.25704 (7)	0.0802 (4)	
O4	0.35180 (13)	0.31127 (18)	0.16290 (6)	0.0711 (4)	

### Atomic displacement parameters (Å^2^)

**Table:**

	*U*^11^	*U*^22^	*U*^33^	*U*^12^	*U*^13^	*U*^23^
C1	0.0566 (9)	0.0505 (9)	0.0424 (8)	−0.0007 (7)	−0.0024 (7)	0.0057 (7)
C2	0.0450 (8)	0.0486 (8)	0.0387 (7)	0.0017 (6)	0.0054 (6)	−0.0016 (6)
C3	0.0599 (10)	0.0615 (10)	0.0477 (9)	0.0048 (8)	−0.0056 (7)	0.0005 (8)
C4	0.0548 (10)	0.0810 (13)	0.0565 (10)	−0.0004 (9)	−0.0112 (8)	−0.0104 (9)
C5	0.0542 (10)	0.0704 (11)	0.0611 (11)	−0.0158 (8)	0.0046 (8)	−0.0129 (9)
C6	0.0598 (10)	0.0564 (10)	0.0524 (9)	−0.0108 (8)	0.0066 (8)	0.0012 (8)
C7	0.0429 (7)	0.0521 (9)	0.0388 (7)	−0.0026 (6)	0.0044 (6)	−0.0012 (6)
C8	0.0527 (9)	0.0446 (8)	0.0472 (8)	0.0008 (7)	0.0028 (7)	0.0060 (7)
C9	0.0425 (8)	0.0404 (8)	0.0496 (9)	0.0035 (6)	0.0038 (6)	0.0074 (6)
C10	0.0487 (9)	0.0499 (9)	0.0610 (10)	0.0058 (7)	0.0096 (8)	0.0053 (8)
C11	0.0764 (13)	0.0988 (16)	0.0972 (16)	0.0140 (12)	0.0463 (12)	0.0203 (13)
C12	0.0402 (7)	0.0468 (8)	0.0478 (8)	0.0013 (6)	−0.0009 (6)	0.0050 (7)
C13	0.0474 (8)	0.0473 (9)	0.0556 (9)	−0.0004 (7)	0.0074 (7)	0.0001 (7)
C14	0.0465 (9)	0.0818 (13)	0.0711 (12)	0.0038 (9)	0.0042 (8)	−0.0141 (10)
C15	0.0447 (10)	0.0960 (17)	0.134 (2)	−0.0010 (10)	0.0160 (12)	−0.0333 (16)
C16	0.0896 (18)	0.0783 (16)	0.172 (3)	−0.0038 (13)	0.0828 (19)	−0.0029 (17)
C17	0.123 (2)	0.1042 (19)	0.128 (2)	0.0311 (17)	0.0813 (19)	0.0423 (17)
C18	0.0792 (13)	0.0869 (14)	0.0748 (13)	0.0201 (11)	0.0293 (11)	0.0233 (11)
N1	0.0582 (8)	0.0473 (8)	0.0453 (7)	−0.0023 (6)	0.0031 (6)	0.0017 (6)
O1	0.0836 (9)	0.0544 (7)	0.0620 (8)	−0.0171 (6)	−0.0088 (6)	0.0092 (6)
O2	0.0628 (7)	0.0523 (7)	0.0563 (7)	−0.0121 (5)	−0.0151 (5)	0.0150 (5)
O3	0.0425 (7)	0.1103 (11)	0.0870 (9)	−0.0043 (7)	0.0093 (6)	−0.0138 (8)
O4	0.0638 (8)	0.0893 (9)	0.0651 (8)	0.0080 (7)	0.0243 (6)	0.0060 (7)

### Geometric parameters (Å, º)

**Table:**

C1—N1	1.2553 (19)	C10—O4	1.334 (2)
C1—C2	1.455 (2)	C11—O4	1.437 (2)
C1—H1	0.9300	C11—H11A	0.9600
C2—C3	1.388 (2)	C11—H11B	0.9600
C2—C7	1.393 (2)	C11—H11C	0.9600
C3—C4	1.369 (2)	C12—C13	1.459 (2)
C3—H3	0.9300	C12—H12	0.9300
C4—C5	1.369 (3)	C13—C18	1.380 (2)
C4—H4	0.9300	C13—C14	1.390 (2)
C5—C6	1.380 (2)	C14—C15	1.376 (3)
C5—H5	0.9300	C14—H14	0.9300
C6—C7	1.383 (2)	C15—C16	1.371 (4)
C6—H6	0.9300	C15—H15	0.9300
C7—O2	1.3604 (17)	C16—C17	1.370 (4)
C8—O2	1.4356 (17)	C16—H16	0.9300
C8—C9	1.495 (2)	C17—C18	1.368 (3)
C8—H8A	0.9700	C17—H17	0.9300
C8—H8B	0.9700	C18—H18	0.9300
C9—C12	1.330 (2)	N1—O1	1.4019 (17)
C9—C10	1.484 (2)	O1—H1A	0.8200
C10—O3	1.1976 (19)		
			
N1—C1—C2	122.37 (14)	O4—C10—C9	111.86 (14)
N1—C1—H1	118.8	O4—C11—H11A	109.5
C2—C1—H1	118.8	O4—C11—H11B	109.5
C3—C2—C7	118.05 (14)	H11A—C11—H11B	109.5
C3—C2—C1	123.16 (14)	O4—C11—H11C	109.5
C7—C2—C1	118.78 (12)	H11A—C11—H11C	109.5
C4—C3—C2	121.30 (16)	H11B—C11—H11C	109.5
C4—C3—H3	119.4	C9—C12—C13	128.71 (14)
C2—C3—H3	119.4	C9—C12—H12	115.6
C5—C4—C3	119.87 (15)	C13—C12—H12	115.6
C5—C4—H4	120.1	C18—C13—C14	118.13 (17)
C3—C4—H4	120.1	C18—C13—C12	118.30 (15)
C4—C5—C6	120.67 (16)	C14—C13—C12	123.54 (15)
C4—C5—H5	119.7	C15—C14—C13	120.4 (2)
C6—C5—H5	119.7	C15—C14—H14	119.8
C5—C6—C7	119.28 (16)	C13—C14—H14	119.8
C5—C6—H6	120.4	C16—C15—C14	119.9 (2)
C7—C6—H6	120.4	C16—C15—H15	120.0
O2—C7—C6	124.41 (14)	C14—C15—H15	120.0
O2—C7—C2	114.76 (13)	C17—C16—C15	120.3 (2)
C6—C7—C2	120.82 (13)	C17—C16—H16	119.9
O2—C8—C9	105.98 (12)	C15—C16—H16	119.9
O2—C8—H8A	110.5	C18—C17—C16	119.7 (2)
C9—C8—H8A	110.5	C18—C17—H17	120.2
O2—C8—H8B	110.5	C16—C17—H17	120.2
C9—C8—H8B	110.5	C17—C18—C13	121.3 (2)
H8A—C8—H8B	108.7	C17—C18—H18	119.3
C12—C9—C10	117.02 (14)	C13—C18—H18	119.3
C12—C9—C8	123.97 (14)	C1—N1—O1	112.30 (12)
C10—C9—C8	119.01 (14)	N1—O1—H1A	109.5
O3—C10—O4	122.93 (16)	C7—O2—C8	119.38 (12)
O3—C10—C9	125.20 (16)	C10—O4—C11	116.56 (16)
			
N1—C1—C2—C3	−1.8 (3)	C10—C9—C12—C13	177.38 (14)
N1—C1—C2—C7	177.49 (15)	C8—C9—C12—C13	−2.3 (2)
C7—C2—C3—C4	0.5 (2)	C9—C12—C13—C18	146.92 (19)
C1—C2—C3—C4	179.80 (17)	C9—C12—C13—C14	−35.1 (3)
C2—C3—C4—C5	−1.1 (3)	C18—C13—C14—C15	−4.1 (3)
C3—C4—C5—C6	0.6 (3)	C12—C13—C14—C15	177.96 (17)
C4—C5—C6—C7	0.5 (3)	C13—C14—C15—C16	0.5 (3)
C5—C6—C7—O2	177.67 (16)	C14—C15—C16—C17	2.7 (4)
C5—C6—C7—C2	−1.1 (3)	C15—C16—C17—C18	−2.3 (4)
C3—C2—C7—O2	−178.30 (14)	C16—C17—C18—C13	−1.4 (4)
C1—C2—C7—O2	2.4 (2)	C14—C13—C18—C17	4.6 (3)
C3—C2—C7—C6	0.6 (2)	C12—C13—C18—C17	−177.4 (2)
C1—C2—C7—C6	−178.70 (15)	C2—C1—N1—O1	179.94 (14)
O2—C8—C9—C12	97.38 (17)	C6—C7—O2—C8	8.8 (2)
O2—C8—C9—C10	−82.28 (16)	C2—C7—O2—C8	−172.33 (14)
C12—C9—C10—O3	0.7 (2)	C9—C8—O2—C7	−173.35 (13)
C8—C9—C10—O3	−179.59 (16)	O3—C10—O4—C11	−1.1 (3)
C12—C9—C10—O4	−178.71 (14)	C9—C10—O4—C11	178.38 (15)
C8—C9—C10—O4	1.0 (2)		

### Hydrogen-bond geometry (Å, º)

**Table:**

*D*—H···*A*	*D*—H	H···*A*	*D*···*A*	*D*—H···*A*
O1—H1*A*···N1^i^	0.82	2.06	2.7836 (19)	146
C15—H15···O3^ii^	0.93	2.53	3.300 (2)	140

Symmetry codes: (i) −*x*, −*y*+2, −*z*; (ii) *x*−1, *y*, *z*.
